# P-1690. Evaluation of an Ultrasensitive Urine LAM Assay for Tuberculosis Using Optofluidic Single-Molecule Counting Technology

**DOI:** 10.1093/ofid/ofaf695.1864

**Published:** 2026-01-11

**Authors:** Cathy Le, Tiffany Truong, Justin Nguyen, Harisha Ramachandraiah, Niamh Nolan, Renee Tobias, Frank Zaugg, Peter Wagner, Valerie Brachet, Johanna Sandlund

**Affiliations:** Fluxus, Inc., Sunnyvale, California; Fluxus, Inc., Sunnyvale, California; Fluxus, Inc., Sunnyvale, California; Foundation for New Innovative Diagnostics, Geneva, Geneve, Switzerland; Fluxus, Inc., Sunnyvale, California; Fluxus, Inc., Sunnyvale, California; Fluxus, Inc., Sunnyvale, California; Fluxus, Inc., Sunnyvale, California; Fluxus, Inc., Sunnyvale, California; Fluxus, Inc., Sunnyvale, California

## Abstract

**Background:**

Sputum-based diagnostics for tuberculosis (TB) are limited in sensitivity, accessibility, and applicability—particularly in children, people living with HIV (PLHIV), and patients with extrapulmonary TB. Urine lipoarabinomannan (LAM) is a well-established TB biomarker, but existing tests are restricted to PLHIV and offer limited sensitivity (LoD ∼250 pg/mL). Fluxus’ single-molecule counting platform integrates optics and microfluidics to enable ultrasensitive detection of proteins in clinical samples. We present preliminary analytical and clinical performance data for a prototype urine LAM assay developed using this platform.

**Methods:**

Analytical characterization included determination of limits of detection (LoD) and quantification (LoQ), dilution linearity, spike recovery, intra- and inter-assay precision, and crossreactivity. Preliminary clinical performance will be evaluated on biobanked urine samples from a cohort of adults with suspected TB, and LAM concentrations will be compared to a TB microbiologic reference standard.

**Results:**

The assay demonstrated LoD and LoQ of 0.068 and ∼2 pg/mL, respectively, with a >5-log dynamic range. Spike recovery in urine averaged 106% (range 80%–130%) and dilution linearity was maintained across 0.01–100 ng/mL (mean 102%, range 86%–134%). Intraassay CV averaged 7.4%, and interassay CV was < 15% across the quantifiable range. No crossreactivity was observed with *Mycobacterium smegmatis* LAM. Testing in clinical samples is ongoing.

**Conclusion:**

The Fluxus ultrasensitive LAM assay offers sub-pg/mL sensitivity, strong linearity, and reproducibility, enabling accurate quantification of LAM in urine across a broad range. Clinical testing in a diverse cohort is ongoing. There is a potential for Fluxus’ single-molecule counting technology to transform non-sputum-based TB diagnostics and support broader use of LAM testing.

**Disclosures:**

Cathy Le, BS, Fluxus: Employee Tiffany Truong, MS, Fluxus: Employee Justin Nguyen, BS, Fluxus: Employee|Fluxus: Employee Niamh Nolan, MS, Fluxus: Advisor/Consultant|Fluxus: Advisor/Consultant Renee Tobias, MS, Fluxus: Employee Frank Zaugg, PhD, Fluxus: Employee Peter Wagner, PhD, Fluxus: Employee Valerie Brachet, PhD, Fluxus: Employee Johanna Sandlund, MD, PhD, Fluxus: Employee

